# Supplementary material for: The dynamic evolution of panarthropod germ cell specification mechanisms
Source: Development. 2026 Apr 14;153(7):dev205168. doi: 10.1242/dev.205168 (PMC13120685; doi:10.1242/dev.205168)
Supplement: Supplementary information [file develop-153-205168-s1.pdf]

Supplementary Materials and Methods

Throughout these Methods, custom scripts (blue text) and output files and folders (magenta text) described are located at the github repository for this study, https://github.com/rishabhrajkapoor/panarthropoda_gc_specification_evolution, commit ID ff9945c. Analysis packages used are indicated in green text. Publicly available scripts used are indicated in gold text.

Inferring germ cell specification mechanisms from published literature

For several species, experimental manipulations have provided direct evidence for the use of either germ plasm or induction (Table S1). For other species with only observational data available in published literature, we inferred the likely mechanism of specification for the ancestral character reconstruction based on patterns in the developmental timing and location of germ cell origination. For species designated as Category 1, with germ cells forming in the early blastoderm and cleavage stages, we inferred that they were using germ plasm. For species designated as Category 3, with germ cells forming late in embryogenesis from the mesoderm, we inferred that they were using induction. For Category 2 species, we inferred the use of germ plasm for species in which granules with the characteristic morphological and spatiotemporal appearance of germ granules (e.g. Eddy, 1975) were reported; otherwise, we inferred induction. Our inferred mechanisms are available in Table S1 and in all_species_annotated.tsv.

Phylogram construction

To visualize the phylogenetic relationships among the 211 species in our dataset of germ cell specification data (Table S1), we concatenated published phylogenies from 23 pre-existing studies of various panarthropod lineages (Table S2) (using notebook phylogram_constraints.ipynb, output file constraint_trees/all_combined_species.nwk). Newick trees were obtained directly from the published studies, from the TimeTree (Kumar et al., 2022) or from the Open Tree of Life (OpenTreeOfLife et al., 2019) websites when available, or were manually extracted from published figures. Individual Newick trees are available in the "constraint_trees" directory. Phylogenetic relationships were delimited up to the level of taxonomic family, except for the families Chrysomelidae, Culicidae, and Drosophilidae, which were further resolved to the subfamily, genus, and species levels, respectively, due to the greater representation of these families in our dataset. We used polytomies to represent intrafamily relationships, paraphyly in inter-family relationships, or unresolved nodes in the arthropod phylogeny. Phylograms were taxonomically annotated with the ete3 package (Huerta-Cepas et al., 2016), manually concatenated, and visualized in the iTOL web server (Letunic and Bork,

2024). Branch lengths in the resulting phylograms (Figures 2-3, Figure S1) are arbitrary and do not reflect evolutionary divergence times.

Ancestral character reconstruction via maximum parsimony

Ancestral character reconstruction of inferred germ cell specification modes (GP: germ plasm; IND: induction) via parsimony was performed on the above phylogram with the GUI interface of Mesquite version 4.0 using default parameters (Figure S1; see Table S3 for hypothesized transitions; input nexus file with GP/IND annotations available at GC_mech_parsimony_input.nex) (https://www.mesquiteproject.org; version 4.0). The results inferred that the ancestral panarthropod specification mode was induction, with a minimum of 24 changes over the history of the panarthropods. When considering all equally parsimonious character histories, changes from induction to maternal inheritance (min = 18, max = 23, average = 20.5) were the most common, but reverse transitions were also inferred (min = 2, max = 7, average = 4.5). At a minimum, reversions from maternal inheritance to induction were inferred in the honeybee *A. mellifera* (Hymenoptera) and the drain fly *C. albipunctata* (Diptera). We note that all maximum parsimony reconstructions assign induction as the ancestral state of Holometabola (Figure S1, node 11), which requires many fewer total transitions within Holometabola (N = 12) than would be required if germ plasm evolved a single time in the last common ancestor of Holometabola (N = 20) as previously proposed (Ewen-Campen et al., 2013a; Lynch et al., 2011) .

Maximum likelihood phylogenetic tree inference

Ancestral character reconstruction requires as an input a phylogenetic tree in which branch lengths are proportional to evolutionary divergence (executed with time_calibrated_busco_phylogenomics.ipynb). To obtain genomic data for phylogenetic inference, we searched NCBI for genomes or transcriptome shotgun assemblies (TSAs) corresponding to our taxa of interest, filtering by >50% BUSCO (Manni et al., 2021) single copy complete ortholog completeness (with arthropoda_odb12, using the "Download and prep protein datasets" portion of time_calibrated_busco_phylogenomics.ipynb) (Table S4). We selected sequence data from exact species matches when available (N = 77 species), else substituted sequence data from a different species in the same taxonomic family (N = 45 substitutions) or order (N = 2 substitutions) when the germ cell specification mechanism was the same for all species in the family or order respectively in our dataset (Table S1). In the case of a substitution, the leaf name in the phylogenetic tree was replaced by the name of the family or order shared with the species in our dataset (Figure S2).

We used BUSCO version 5.8.3 with the arthropoda_odb12 set (Manni et al., 2021) to extract single-copy protein ortholog sequences. Upstream of BUSCO, protein predictions were generated for TSAs using TransDecoder version 5.7.1 (using

Development • Supplementary information

transdecoder.sh and run_busco_prot_tsa.sh) (Haas et al., 2013) or extracted from .fa files for annotated genomes in NCBI, selecting the longest splice isoform per gene (using the "Download and prep protein datasets" portion of time_calibrated_busco_phylogenomics.ipynb and run_busco_annotated_genome.sh). For unannotated genes, protein sequences were extracted directly from nucleotide fasta files using BUSCO with the "-m genome" flag. BUSCO protein sequences were submitted to the BUSCO_phylogenomics pipeline (release 20240919) (https://github.com/jamiemcg/BUSCO_phylogenomics) with default parameters to generate a concatenated supermatrix of trimmed MUSCLE alignments (Edgar, 2004) (output supermatrix: BUSCO_py/supermatrix/SUPERMATRIX.phylip).

Deep-branching panarthropod relationships have been comprehensively examined by several prior studies and are often susceptible to reconstruction artifacts (Giribet, 2018; Meusemann et al., 2020). We therefore used the same literature-derived tree topologies (Table S2) used for phylogram construction to constrain the family, order, class, and phylum-level relationships in our trees when these ranks had previously been shown to be monophyletic (constraint tree at BUSCO_py/constraint_tree_iqtree_species.tree). Polytomies in our constraint tree were resolved via maximum likelihood inference from our supermatrix with IQ-Tree version 2.4 (model: LG+G4, with per-gene partitions) (Minh et al., 2020) (executed with run_iqtree_constrained_search.sh, outputs in BUSCO_py/supermatrix with the "constrained_optimized" prefix). The resulting maximum likelihood tree was rooted with Tardigrada as an outgroup (Howard et al., 2022).

Time calibration

Approximate likelihood molecular clock calibrations of our IQ-Tree maximum likelihood tree were performed using the IQ2MC workflow (Demotte et al., 2025), which integrates IQ-Tree version 3.0.1 (Wong et al., 2025) with MCMCTree in PAML version 4.10.9 (Yang, 1997; Yang, 2007) (using time_calibrated_busco_phylogenomics.ipynb). The fossil calibrations were derived from references listed in Table S5, including (Wiegmann et al., 2011) for Diptera, (Misof et al., 2014) for non-dipteran insects, (Bernot et al., 2023) for non-insect Pancrustacea, and (Howard et al., 2022) for Chelicerata and the internal nodes of the panarthropod phylogeny (rooted constraint tree available at BUSCO_py/supermatrix/fossil_rooted_constrained_optimized.treefile). Hessian matrices and gradient vectors were obtained using IQ-Tree with the "-mcmctree" flag, the pre-computed supermatrix and maximum-likelihood tree, an LG+G4 substitution model, and a single partition to promote computational efficiency (using iqtree3_iq2mcmc.sh, outputs available at BUSCO_py/supermatrix/ with prefix "mcmc_prep_no_partition"). MCMCTree was then run with an independent rates relaxed molecular clock, burnin = 20000, sampfreq = 100 and nsample = 20000. Convergence of inferred divergence times was confirmed via two independent runs of MCMCTree

Development • Supplementary information

(PAML control files at BUSCO_py/supermatrix/run1R_mcmc_prep_no_partition.mcmctree.ctl and BUSCO_py/supermatrix/run2R_mcmc_prep_no_partition.mcmctree.ctl, output files in the same directory with mcmctree.out suffix, selected time-calibrated Newick tree at mcmctree.tree).

Maximum likelihood ancestral character reconstruction

We used the fossil-calibrated phylogeny of 124 species to infer ancestral states via marginal maximum likelihood using the phytools package (Revell, 2024) (using maximum_likelihood_ancestral_reconstruction.R). Five *Mk* substitution models were fit to the data, including models allowing for gamma heterogeneity in rates among branches or hidden rate transitions to account for possibly divergent evolutionary regimes under the deep time scales analyzed in our data (Table S6). The best-fitting model by the Akaike information criterion was the symmetric rates model with gamma-rates heterogeneity (Table S6), suggesting that although the overall rate of transition from germ plasm to induction is equivalent to the rate of the reverse transition, the value of this single rate parameter shows variation over the phylogeny (Revell and Harmon, 2024). The equivalent rates of transition to and from germ plasm are consistent with the reversions to induction noted in the parsimony analysis above, although we note that the quantification of evolutionary rate was only possible with our time-calibrated phylogeny. The rate heterogeneity is consistent with the observation via maximum parsimony of 15/24 transitions in Insecta alone (Figure S1). Inferred transitions with support from maximum likelihood are reported in Table S3.

Inferring germ band development type from published literature

We classified species by germ band type based on embryological descriptions as follows: in short germ band embryos, the initial germ band comprises only head segments; in intermediate germ band embryos, the initial germ band comprises the head and thorax; and in long germ band embryos, the initial germ band comprises head, thorax and abdominal segments.

Testing the relationship between germ band development type and germ cell specification mode

To test the hypothesis that long vs. short germ band development (Krause, 1939) influences the evolution of germ plasm in insects, we first performed phylogenetic logistic regression (Ives and Theodore, 2010) with phylolm (version 2.6.5 phylolm, method = logistic_MPLE), in which the independent variable was germ band type (three-level categorical predictor with “short” as the reference level and “long” and “intermediate” as two alternatives) and germ cell specification mode was the dependent variable (induction = 0, germ plasm = 1; Figure S3) (using germ_band_correlations.R,

Development • Supplementary information

with the phylogeny in insect_only.tree and data in insect_germ_band.tsv). We found a statistically significant association between long germ band development and germ plasm (coefficient = 3.02, p-value = 2.81e−05) and no such association between intermediate germ band development and germ plasm (coefficient = −0.01, p-value = 0.99).

Since Pagel's test (Pagel, 1994) is performed on binary variables, we recoded all "intermediate" germ band annotations as "short" to produce a binary trait (using germ_band_correlations.R). The null model assumes that the rate of evolution of germ cell development mode is independent of the transition in long vs. short germ band development. Our three alternative hypotheses assume either that germ band type and germ cell specification mode mutually influence each other's evolution ("bidirectional"), or that germ band type influences germ cell specification mode but not vice versa ("GC dependent"), or that germ cell specification mode influences germ band type but not vice versa ("GB dependent"). Maximum likelihood rate fits for the bidirectional model are shown in Figure S4.

All Pagel covariation models assuming coupled evolutionary rates among these two characters are significantly better fits to the data than the null model of independent evolution (likelihood ratio test p-value < 1e−3; Figure S4; Table S8). We note that the bidirectional and GB-dependent models perform similarly to the GC-dependent model.

Search for oskar *orthologs*

We searched the genomes and transcriptomes listed in Table S4 using hmmsearch (http://eddylab.org/software/hmmer/Userguide.pdf) with the consensus profile HMMs for the LOTUS (LOTUS_CONSENSUS.hmm) and OSK (OSK_CONSENSUS.hmm) domains as previously defined (Blondel et al., 2021) (using oskar_search.ipynb, hmmsearch outputs are in the hmmsearch_results folder). For this *oskar* ortholog search, a chromosome-level, unannotated genome assembly (GCA_965637365.1) for *Clogmia albipunctata* that became available at the time of performing this analysis was substituted for the genome assembly for this species that is listed in Table S4 (GCA_022818195.1). Following our previous approach (Blondel et al., 2021), we defined Oskar orthologs as proteins that were hmmsearch (E-value < 0.05) hits to both the LOTUS and OSK domains. Amino acid sequences of all detected Oskar proteins are available in oskar_proteins.fa file b.

For unannotated genomes, we first obtained *ab initio* protein predictions using the AUGUSTUS webserver with default parameters (Stanke et al., 2004). Pre-trained species parameters for gene model prediction were selected from close phylogenetic relatives in the AUGUSTUS web server as follows: *Drosophila melanogaster* for *C. albipunctata* (GCA_022818195.1) and *Mayetiola destructor* (GCA_000149185.1); *Nasonia vitripennis* for *Macrocentrus cingulum* (GCA_045786645.1); *Heliconius melpomene* for *Epiphyas postvittana* (GCA_964304675.1) and *Nymphalis antiopa*

Development • Supplementary information

(GCA_964258955.1); *Rhodnius prolixus* for *Pyrrhocoris apterus* (GCA_039877355.1); and *Tribolium castaneum* for *Tribolium confusum* (GCA_029207805.1).

Due to the unique position of *C. albipunctata* (family: Psychodidae) as the sole dipteran lacking germ plasm in our dataset, and its lacking a published annotated genome, we additionally tested the hypothesis that this species' genome lacks *oskar* via tblastn (blast version 2.9.0) with the *Phlebotomus papatasi* (family: Psychodidae) Oskar protein (XP_055701593.1) as a query (using oskar_search.ipynb). We found no full-length tblastn hits but did find a possible hit to the OSK domain with evidence of multiple in-frame stop codons (Figure S5), consistent with the hypothesis of pseudogenization of *oskar* in *C. albipunctata*.

Testing the relationship between oskar presence and germ cell specification mode

Since gene loss in metazoans is thought to be irreversible (Elmer and Clobert, 2025) and Pagel's test (Pagel, 1994) permits bidirectional state transitions, we opted only for phylogenetic logistic regression with phylolm (method logistic_MPLE) to test the association between germ cell specification mode (dependent variable, 1 = germ plasm, 0 = induction) and *oskar* presence (independent variable, 1 = presence, 0 = absence) within insects while accounting for phylogenetic relatedness. We analyzed all 81 insect datasets shown in Table S9, including 24 within-family substitutions when genomic or transcriptomic data were not available for a species (using oskar_fitphyloglm.R with oskar_data.tsv and insect_only.tree as inputs).

We found no association of germ-plasm mode with BUSCO completeness (coefficient = 0.23, p-value = 0.37). However, *oskar* presence (Table S9) was moderately (although not statistically significantly) associated with BUSCO completeness (mean BUSCO completeness for *oskar*-containing datasets = 83.29%, mean BUSCO completeness for *oskar*-absent datasets = 78.09%, Mann-Whitney U p-value = 0.08). We therefore additionally included the z-score normalized BUSCO score as a covariate in our model

The results showed a significant association between *oskar* presence in the genome and the use of germ plasm to specify PGCs (coefficient = 2.58, adjusted odds-ratio: = 13.28, p-value = 0.0006). The association between *oskar* presence and maternal inheritance mode remained strong and statistically significant even after removing 24 within-family substitutions made when neither genomic nor transcriptomic data were available for a species (coefficient = 2.56, adjusted odds ratio = 12.93, p-value = 0.0003).

Tree visualization

Trees were visualized and annotated using the iTOL web server v7 (Letunic and Bork, 2024)**,** except for Figure S1 and Figure S2, which were generated as outputs of Mesquite and phytools, respectively.

Development • Supplementary information

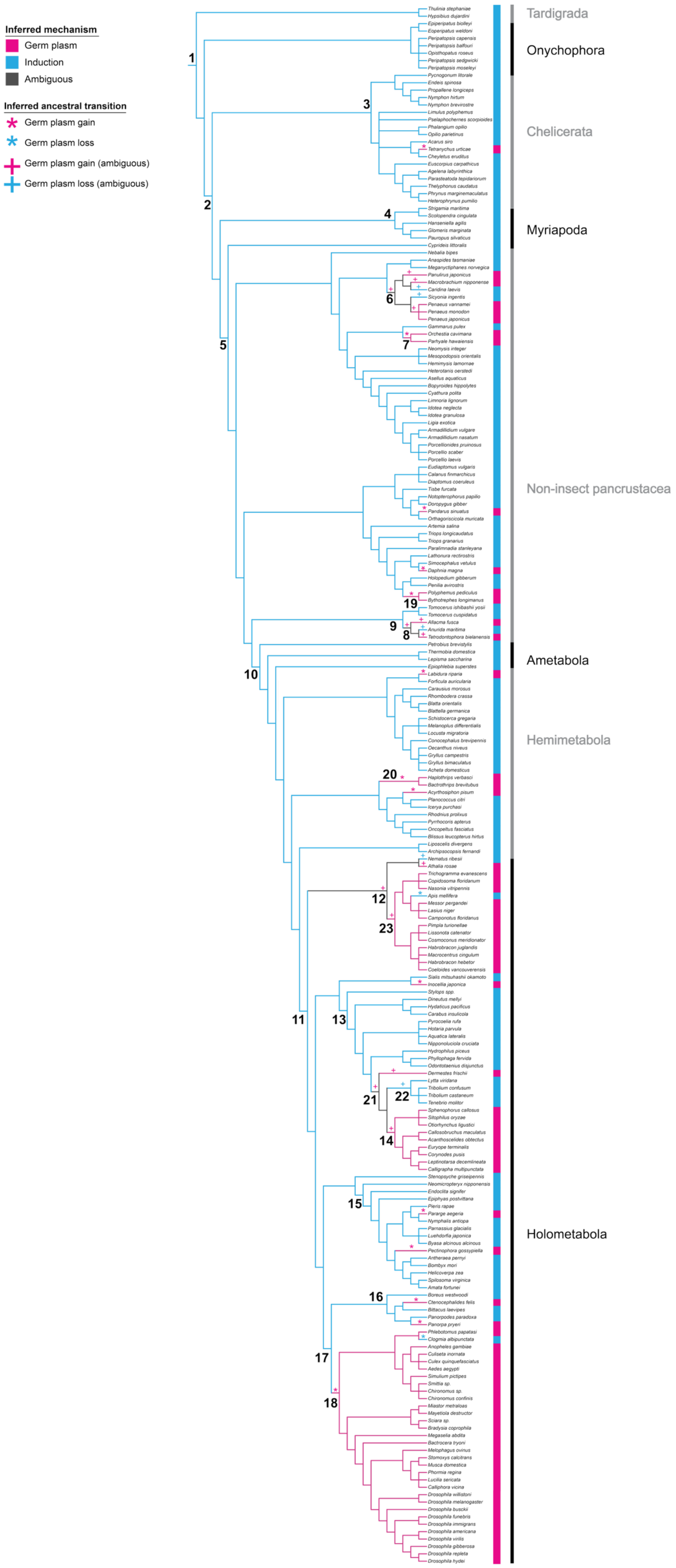

Inferred mechanism
Germ plasm
Induction
Ambiguous
Inferred ancestral transition
Germ plasm gain
Germ plasm loss
Germ plasm gain (ambiguous)
Germ plasm loss (ambiguous)
1
2
3
4
5
6
7
8
9
10
11
12
13
14
15
16
17
18
19
20
21
22
23
Tardigrada
Onychophora
Chelicerata
Myriapoda
Non-insect pancrustacea
Ametabola
Hemimetabola
Holometabola

Fig. S1. Phylogram with maximum parsimony ancestral state inference. Phylogram of the 211 species for which we compiled germ cell specification data in this study (Table S1), with select inner nodes numbered for reference (1 = Panarthropoda; 2 = Arthropoda; 3 = Chelicerata; 4 = Myriapoda; 5 = Pancrustacea; 6 = Decapoda; 7 = Hyalidae + Talitridae; 8 = Poduromorpha + Symphypleona; 9 = Collembola; 10 = Insecta; 11 = Holometabola; 12 = Hymenoptera; 13 = Coleoptera; 14 = Chrysomelidae + Curculionidae; 15 = Lepidoptera; 16 = Mecoptera + Siphonaptera; 17 = Antliophora (Diptera + Mecoptera + Siphonaptera); 18 = Diptera; 19 = Onychopoda; 20 = Thysanoptera; 21 = Meloidae + Tenebrionidae + Dermestidae + Chrysomelidae; 22 = Meloidae + Tenebrionidae). Species are labeled with blue or pink boxes to indicate induction or germ plasm, respectively. Internal branches are colored according to inferred ancestral states, with grey indicating that multiple equally parsimonious ancestral states are possible. Asterisks indicate unambiguous transitions (gains or losses of germ plasm); i.e., no other transition at these nodes would result in a parsimonious character history. Plus (+) signs indicate possible transitions at nodes with ambiguous character states. Note that some ambiguous character histories are mutually exclusive: e.g., one parsimonious scenario for Decapoda (node 6) is a gain of germ plasm at node 6 followed by terminal losses in the branches leading to *Caridina laevis* and *Sicyonia ingentis*, while another equally parsimonious but mutually exclusive scenario would be independent gains in *Panulirus japonicus*, *Macrobrachium nipponense,* and the common ancestor of the genus *Penaeus*.

Development • Supplementary information

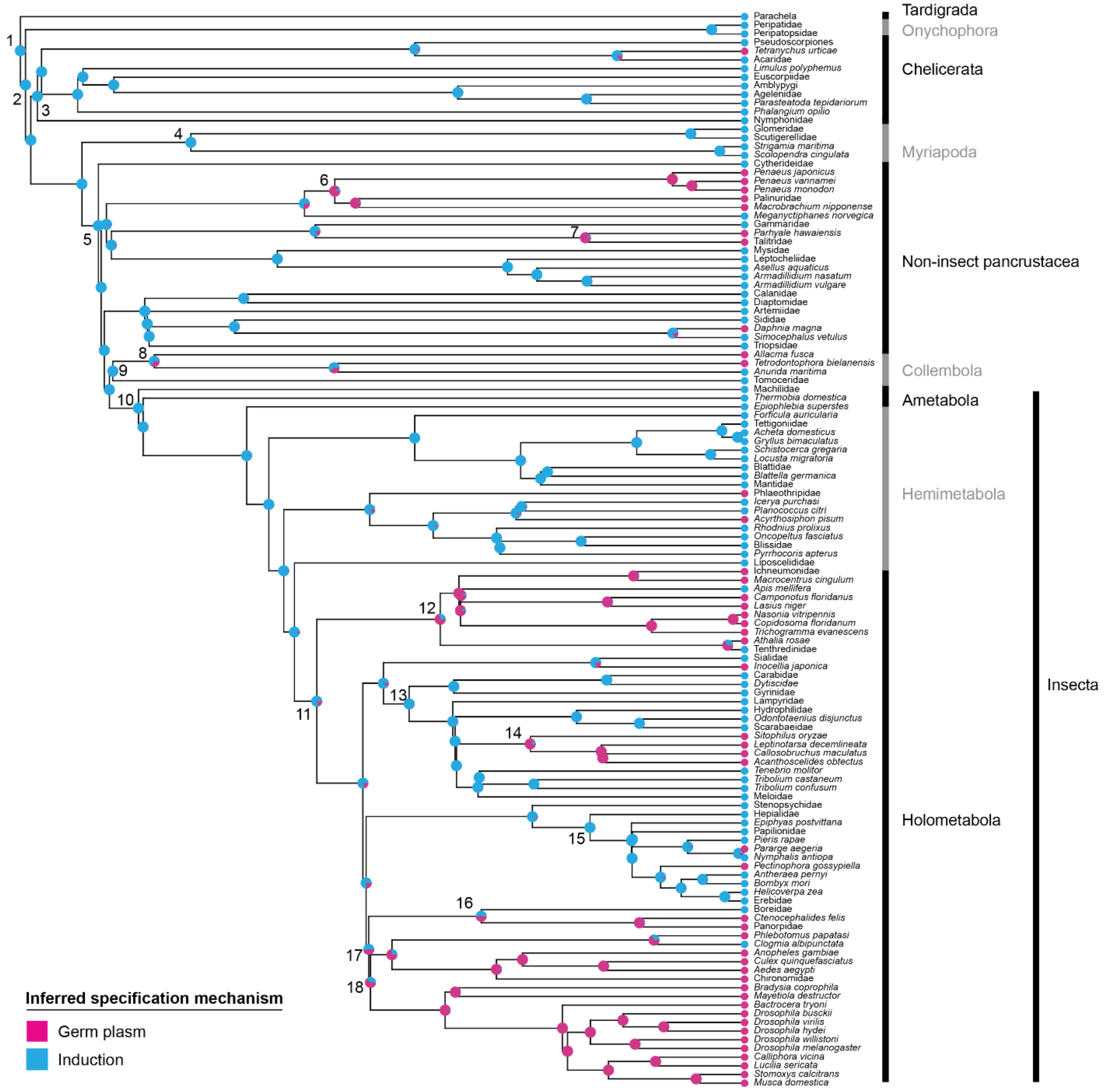

Fig. S2. Maximum likelihood ancestral state reconstruction. Maximum likelihood inference was performed under the symmetric transition model with gamma rate heterogeneity. Tips are labeled with colored circles according to observed induction (blue) or germ plasm (pink) mode. Pie charts at internal nodes report marginal probabilities of induction or germ plasm as the ancestral state. Select ancestral nodes are numbered with clade names and probabilities reported in Table S7. Leaf names at the family or order levels indicate a substitution for tree construction.

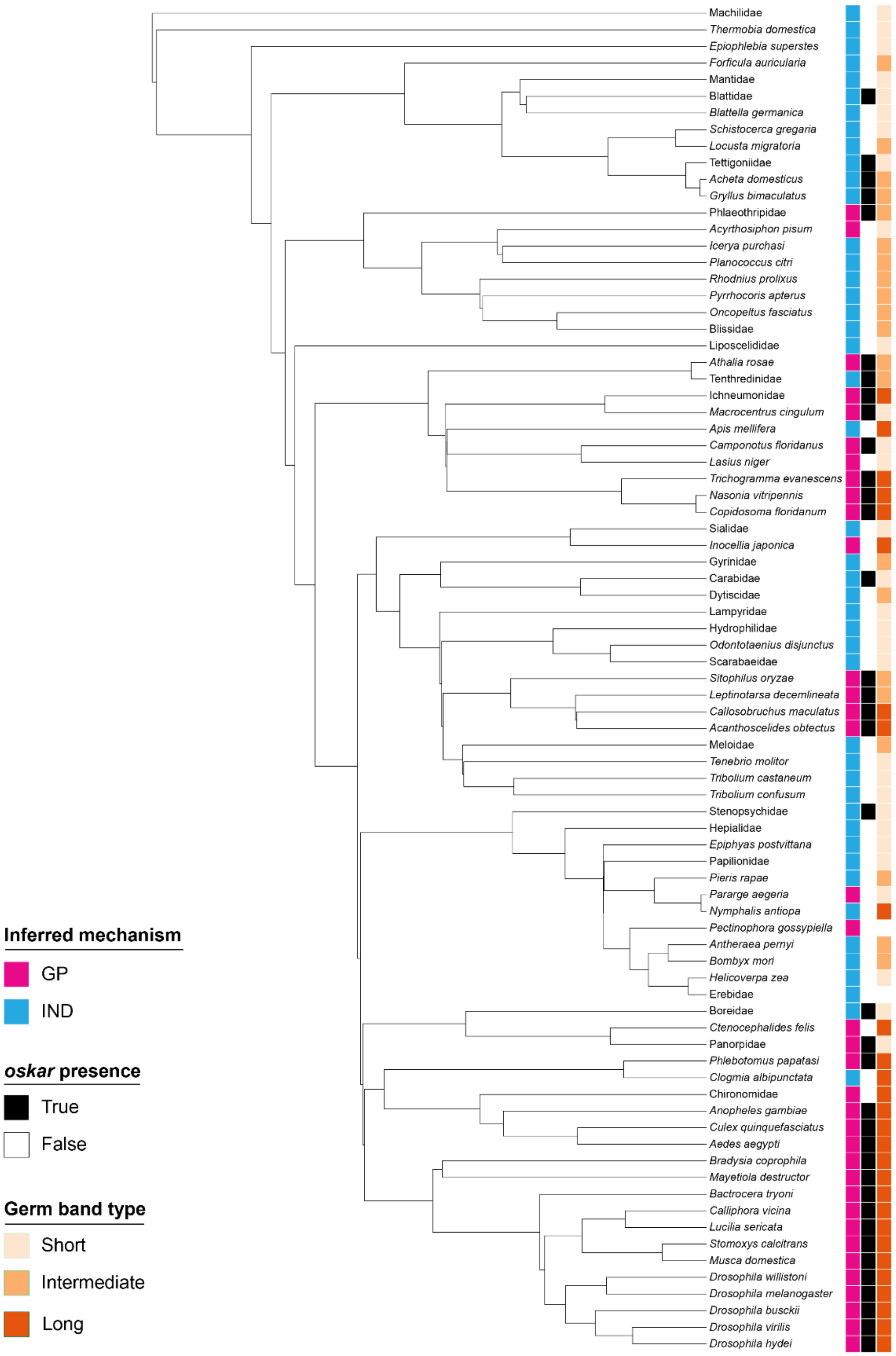

Fig. S3. Relationship between germ cell specification mode (pink/blue), *oskar* presence (black/white), and germ band type (orange shades). Time-calibrated phylogeny of the insect species with genomic/transcriptomic data available at the time of writing (N = 81; Table S9). Leaf names at the family level indicate a substitution for tree construction and inference of *oskar* presence or absence.

Development • Supplementary information

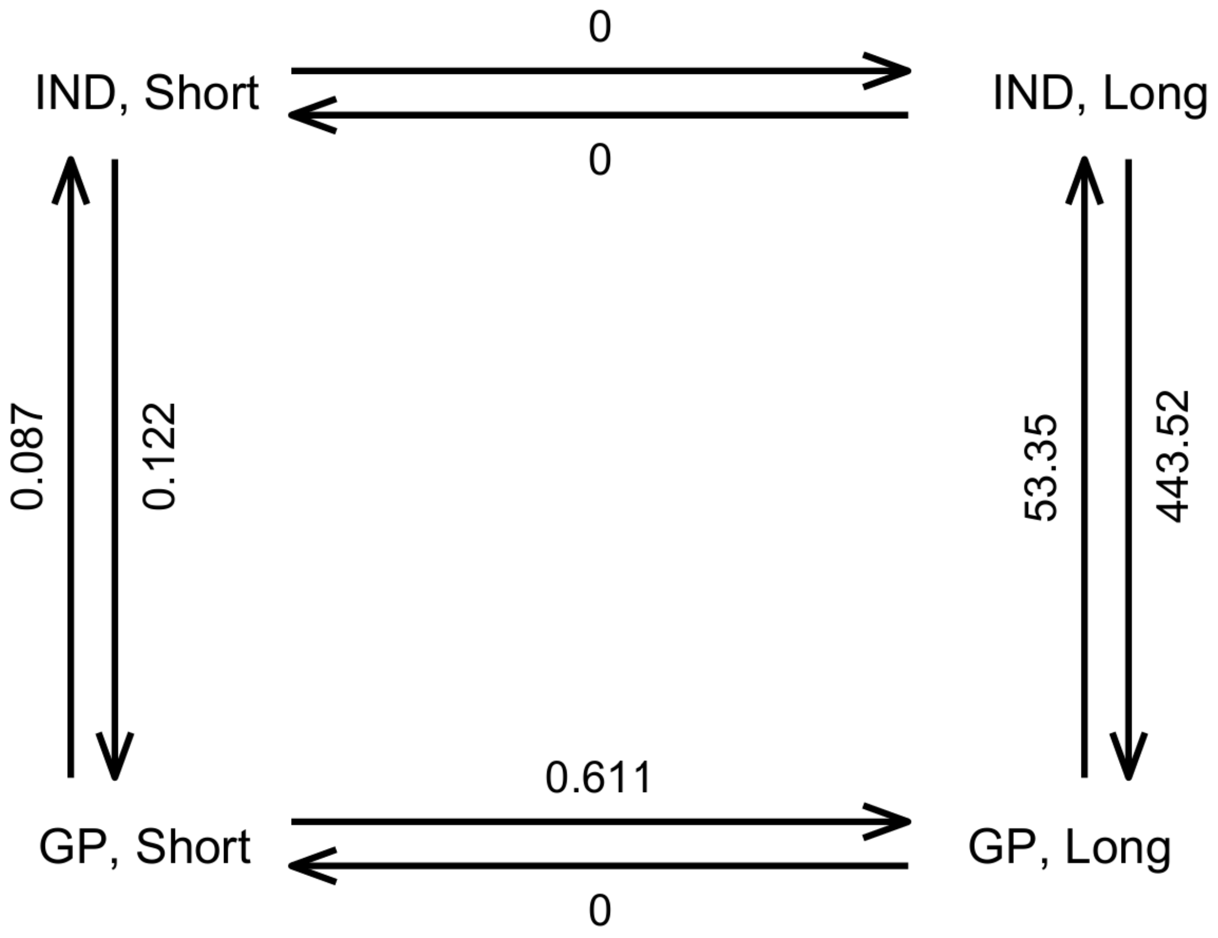

Fig. S4. Pagel test result. Maximum likelihood rate fits for Pagel's test of a bidirectional dependency between transition rates in germ cell specification mode and germ band mode. GP = germ plasm; IND = induction; Short = short germ band; Long = long germ band. A relationship between germ plasm and long germ band modes is supported by the higher transition rate from “GP, Short” to “GP, Long” (0.611) than from “GP, Long” to “GP, Short” (0) or from “IND, Short” to “IND, Long” (0). Likewise, the transition rate from “IND, Long” to “GP, Long” (443.52) is higher than the transition rate from “GP, Long” to “IND, Long” (53.55). The dependent rates model better fits the data than an independent rates model (Table S8).

Development • Supplementary information

```
Query  236       TDYNRLLVLLKEMGIQAIVTTLPPIANHSYLSSMQHNWMKLNVFLRRQPLCIDICEL  292
                 T ++ +L+LL E+     + + P I +    S++Q  W    +F++ Q LC D+C L
Sbjct  42289309  TKWSTILILLIEVFQSGQIFSFPYI*SSLC*SAVQRPWQFPLIFVKCQKLCFDLCSL  42289479
```

Fig. S5. tblastn results of Oskar against the *Clogmia albipunctata* genome. The sole tblastn hit of *Phlebotomus papatasi* Oskar protein (XP_055701593.1) against the *C. albipunctata* genome (version GCA_965637365.1), showing a partial hit on chromosome 2 (accession OZ281279.1) to the OSK domain.

Development • Supplementary information

Table S1. Data on the timing and mechanism of germ cell origin across Panarthropoda. The "GP?" column designates whether observational (OB) or experimental (EX) data have supported the existence of germ plasm. The "IND?" column designates whether observational (OB) or experimental (EX) data have supported induction as the mechanism of germ cell specification. The "timing" column describes the timing of germ cell origination during embryogenesis and categorizes the timing as (1) blastoderm formation, (2) gastrulation, or (3) mesoderm differentiation. The "location" column describes the spatial position of germ cells when they are first reported as detectable. The "criteria" column lists the nature of the data used to identify germ cells and infer mechanism of specification: LM = light microscopy, TEM = transmission electron microscopy, SEM = scanning electron microscopy, MM = molecular markers, CL = cell lineage tracing, TS = transcriptomic data, EX = experimental manipulations.

order	suborder	family	scientific name	common name	GP?	IND?	timing	location	criteria	reference
**TARDIGRADA**										
Parachela		Hypsibiidae	*Thulinia stephaniae*			EX	(2) cleavage	blastopore	LM, CL, EX (laser ablation)	(Hejnol and Schnabel, 2005)
Parachela		Hypsibiidae	*Hypsibius dujardini*				(2) cleavage	blastopore	LM, MM (*piwi*, *vasa in situ*), CL	(Gabriel et al., 2007; Heikes et al., 2023; Wenck, 1914)
**ONYCHOPHORA**										
		Peripatidae	*Eoperipatus weldoni*				(3) late segmented germ band	posterior germ band (walls of splanchnic mesoderm)	LM	(Evans, 1901)
		Peripatidae	*Epiperipatus biolleyi*				(3) early segmented germ band	posterior germ band	TEM	(Mayer and Tait, 2009)
		Peripatopsidae	*Opisthopatus roseus*				(3) early segmented germ band	posterior germ band	TEM	(Mayer and Tait, 2009)
		Peripatopsidae	*Peripatopsis capensis*				(3) segmented germ band	posterior germ band	LM	(Manton, 1949; Sedgwick, 1887)
		Peripatopsidae	*Peripatopsis balfouri*				(3) segmented germ band	posterior germ band	LM	(Manton, 1949)
		Peripatopsidae	*Peripatopsis moseleyi*				(2) gastrulation	blastopore	LM	(Manton, 1949)
		Peripatopsidae	*Peripatopsis sedgwicki*				(3) segmented germ band	posterior germ band	LM	(Manton, 1949)
**CHELICERATA**										
Pantopoda	Colossendeoidea	Pycnogonidae	*Pycnogonum litorale*				(3) fifth instar	flanking the midgut	LM, TEM, SEM	(Alexeeva and Tamberg, 2021)
Pantopoda	Phoxychilidioidea	Endeidae	*Endeis spinosa*				(3) sixth instar	mesoderm surrounding midgut	LM	(Dogiel, 1913)
Pantopoda	Nymphonoidea	Nymphonidae	*Nymphon hirtum*				(3) sixth instar	mesoderm surrounding midgut	LM	(Dogiel, 1913)
Pantopoda	Nymphonoidea	Nymphonidae	*Nymphon brevirostre*				(3) third instar	flanking the midgut	LM, TEM, SEM	(Alexeeva et al., 2018)
Pantopoda	Nymphonoidea	Callipallenidae	*Propallene longiceps*				(3) third instar	flanking the midgut	LM	(Miyazaki and Makioka, 2012)
Xiphosura		Limulidae	*Limulus polyphemus*	Atlantic horseshoe crab			(3) segmented germ band	dorsal side of coelomic cavities	LM	(Kingsley, 1893)
Trombidiformes		Cheyletidae	*Cheyletus eruditus*				(3) segmented germ band	ventral mesoderm	LM	(Hafiz, 1935)
Trombidiformes		Tetranychidae	*Tetranychus urticae*	red spider mite, two-spotted spider mite	OB		(1) blastoderm	randomly distributed in yolk	LM, MM (*vasa in situ*)	(Dearden et al., 2003)
Sarcoptiformes		Acaridae	*Acarus siro*	flour mite			(3) segmented germ band	behind third larval leg	LM	(Hughes, 1950)
Opiliones	Eupnoi	Phalangiidae	*Opilio parietinus*				(2) early germ band	posterior germ band/blastopore	LM	(Holm, 1947)
Opiliones	Eupnoi	Phalangiidae	*Phalangium opilio*				(2) early germ band	posterior germ band/blastopore	LM, MM (*vasa in situ*)	(Faussek, 1888; Faussek, 1892; Gainett et al., 2022)
Scorpiones		Euscorpiidae	*Euscorpius carpathicus*				(2) early germ band	posterior germ disc/blastopore	LM	(Brauer, 1894)
Pseudoscorpiones		Chernetidae	*Pselaphochernes scorpioides*				(3) segmented germ band	mesoderm surrounding posterior midgut	LM	(Weygoldt, 1964)
Amblypygi		Phrynidae	*Heterophrynus pumilio*				(3) segmented germ band	ventral walls of mesodermal coelomic sacs	LM	(Gough, 1902)
Amblypygi		Phrynidae	*Phrynus marginemaculatus*	spotted tailless whip scorpion			(3) segmented germ band	ventral walls of mesodermal coelomic sacs	LM	(Weygoldt, 1975)
Uropygi		Thelyphonidae	*Thelyphonus caudatus*				(3) segmented germ band	ventral walls of mesodermal coelomic sacs	LM	(Schimkewitsch, 1903)
Araneae	Araneomorphae	Theridiidae	*Parasteatoda tepidariorum*	common house spider			(3) segmented germ band	ventral walls of mesodermal coelomic sacs	LM, MM (*vasa, piwi in situ*)	(Schwager et al., 2015)

Development • Supplementary information

Araneae	Araneomorphae	Agelenidae	*Agelena labyrinthica*				(3) segmented germ band	ventral walls of mesodermal coelomic sacs	LM	(Kautzsch, 1910)
**MYRIAPODA**										
Scolopendromorpha		Scolopendridae	*Scolopendra cingulata*	Mediterranean banded centipede			(2) gastrulation	blastopore	LM	(Heymons, 1901)
Geophilomorpha		Linotaeniidae	*Strigamia maritima*				(2) blastoderm	blastopore	LM, MM (*vasa* and *nanos in situ*)	(Green and Akam, 2014)
Tetramerocerata		Pauropodidae	*Pauropus silvaticus*				(3) germ band	mesoderm	LM	(Tiegs, 1947)
Cephalostigmata		Scutigerellidae	*Hanseniella agilis*				(3) germ band	mesoderm	LM	(Tiegs, 1940)
Glomerida		Glomeridae	*Glomeris marginata*	pill millipede			(3) germ band	mesoderm in what will become the seventh and eighth leg segments as they form	LM	(Dohle, 1964)
**NON-HEXAPOD PANCRUSTACEA**										
Podocopa		Cytherideidae	*Cyprideis torosa*				(2) gastrulation	from the mesoderm shortly after gastrulation	LM	(Weygoldt, 1960)
Calanoida		Calanidae	*Calanus finmarchicus*				(3) nauplius hatchling	from the mesoderm on either side of the intestine at posterior end of nauplius	LM	(Grobben, 1881)
Calanoida		Diaptomidae	*Eudiaptomus vulgaris*				(3) nauplius hatchling	from the mesoderm on either side of the intestine at posterior end of nauplius	LM	(Grobben, 1881)
Siphonostomatoida		Dichelesthiidae	unnamed species				(1) 32-cell stage	blastopore; last cell to bud out from the yolk and first cell to enter during gastrulation	LM	(McClendon, 1907)
Siphonostomatoida		Pandaridae	*Orthagoriscicola muricata*				(1) 32-cell stage	blastopore; last cell to bud out from the yolk and first cell to enter during gastrulation	LM	(McClendon, 1907)
Siphonostomatoida		Pandaridae	*Pandarus sinuatus*				(1) 32-cell stage	blastopore; last cell to bud out from the yolk and first cell to enter during gastrulation	LM	(McClendon, 1907)
Harpacticoida		Tisbidae	*Tisbe furcata*				(1) 64-cell stage	blastopore; last cell to bud out from the yolk and first cell to enter during gastrulation	LM	(Witschi, 1934)
Cyclopoida		Notodelphyidae	*Pachypygus gibber*				(2) gastrulation	blastopore, internalized soon after endoderm	LM	(Schimkewitsch, 1896)
Cyclopoida		Notodelphyidae	*Notopterophorus papilio*				(2) gastrulation	blastopore, internalized soon after endoderm	LM	(Schimkewitsch, 1896)
Leptostraca	Nebaliacea	Nebaliidae	*Nebalia bipes*				(3) late germ band	ventral walls of coelomic sacs	LM	(Manton, 1934)
Mysida		Mysidae	*Hemimysis lamornae*				(2) before gastrulation	center of germ disc/blastopore	LM	(Manton, 1928)
Mysida		Mysidae	*Neomysis integer*				(2) before gastrulation	center of germ disc/blastopore	LM	(Needham, 1937)
Mysida		Mysidae	*Mesopodopsis orientalis*				(2) before gastrulation	along median line of germ disc behind ectoteloblasts	LM	(Nair, 1939)
Tanaidacea		Leptocheliidae	*Heterotanais oerstedii*				(2) gastrulation	blastopore, internalized soon after yolk cells	LM	(Scholl, 1963)
Isopoda	Cymothoida	Bopyridae	*Bopyroides hippolytes*				(3) early germ band	lateral clusters, associated with liver rudiment	LM	(Strömberg, 1971)
Isopoda	Cymothoida	Anthuridae	*Cyathura polita*				(3) early germ band	lateral clusters, associated with liver rudiment	LM	(Strömberg, 1972)
Isopoda	Oniscidea	Ligiidae	*Ligia exotica*	sea roach			(3) just before hatching	last thoracic segment and first two pleonic segments	LM	(Terao and Cheng, 1926)
Isopoda	Oniscidea	Armadillidiidae	*Armadillidium nasatum*				(2) gastrulation	mesendodermal cluster at beginning of gastrulation	LM	(Goodrich, 1939)
Isopoda	Oniscidea	Armadillidiidae	*Armadillidium vulgare*	common pill-bug			(2) gastrulation	mesendodermal cluster at beginning of gastrulation	LM	(Lane, 1977)
Isopoda	Oniscidea	Porcellionidae	*Porcellio laevis*	swift woodlouse			(2) gastrulation	mesendodermal cluster at beginning of gastrulation	LM	(Goodrich 1939)
Isopoda	Oniscidea	Porcellionidae	*Porcellionides pruinosus*				(2) gastrulation	mesendodermal cluster at beginning of gastrulation	LM	(Lane, 1977)
Isopoda	Oniscidea	Porcellionidae	*Porcellio scaber*	common rough woodlouse			(2) gastrulation	mesendodermal cluster at beginning of gastrulation	LM	(Wolff, 2009)
Isopoda	Valvifera	Idoteidae	*Idotea granulosa*				(2) gastrulation	mesendodermal cluster at beginning of gastrulation	LM	(Strömberg, 1965)
Isopoda	Valvifera	Idoteidae	*Idotea neglecta*				(2) gastrulation	mesendodermal cluster at beginning of gastrulation	LM	(Strömberg, 1965)
Isopoda	Limnoriidea	Limnoriidae	*Limnoria lignorum*				(2) gastrulation	mesendodermal cluster at beginning of gastrulation	LM	(Strömberg, 1968)
Isopoda	Asellota	Asellidae	*Asellus aquaticus*	pond slater, water louse			(2) gastrulation	blastopore	LM	(Needham, 1942)
Amphipoda	Senticaudata	Gammaridae	*Gammarus pulex*				(2) gastrulation	mesendodermal cluster at beginning of gastrulation	LM	(Scholtz, 1990)
Amphipoda	Senticaudata	Talitridae	*Orchestia cavimana*				(1) 8-cell stage	*a* micromere	LM, CL	(Scholtz and Wolff, 2002; Wolff and Scholtz, 2002)
Amphipoda	Senticaudata	Hyalidae	*Parhyale hawaiensis*	sand flea	EX	EX	(1) 8-cell stage	*g* micromere (note: evidence that germ line can also regenerate)	LM, MM (*vasa*, *gcl*, *orb in situ*; Vasa antibody), EX (cell ablation)	(Extavour, 2005; Gupta and Extavour, 2013; Kaczmarczyk, 2014; Özhan-Kizil et al., 2009)
Anaspidacea		Anaspididae	*Anaspides tasmaniae*	mountain shrimp			(3) shortly before	ventral walls of mesodermal coelomic sacs in T1/T2	LM	(Hickman, 1936)

							hatching			
Euphausiacea		Euphausiidae	*Meganyctiphanes norvegica*	northern krill	OB		(1) 32-cell stage	X_d and X_v cells, sister to endoderm	LM, CL	(Alwes and Scholtz, 2004; Taube, 1909; Taube, 1915)
Decapoda	Dendrobranchiata	Sicyoniidae	*Sicyonia ingentis*	ridgeback prawn	OB		(2) gastrulation	MEvpp cell (medial ventral mesoderm)	LM, CL	(Hertzler, 2002; Pawlak et al., 2010)
Decapoda	Dendrobranchiata	Penaeidae	*Penaeus vannamei*	Pacific white shrimp, whiteleg shrimp	OB		(2) gastrulation	$X_v$pp cell (ventral posterior mesendoderm)	LM, CL	(Hertzler, 2005; Pawlak et al., 2010)
Decapoda	Dendrobranchiata	Penaeidae	*Penaeus monodon*	black tiger shrimp	OB		(2) gastrulation	$X_v$pp cell (ventral posterior mesendoderm)	LM, CL	(Biffis et al., 2009)
Decapoda	Dendrobranchiata	Penaeidae	*Penaeus japonicus*	Kuruma shrimp; Japanese tiger shrimp	OB		(2) gastrulation	$X_v$pp cell (ventral posterior mesendoderm)	LM, CL, TEM	(Grattan et al., 2013; Pawlak et al., 2010; Vincent and Hertzler, 2018)
Decapoda	Pleocyemata (Caridea)	Atyidae	*Caridina laevis*				(3) segmented embryo	ventral mesoderm on either side of the gut	LM	(Nair, 1949)
Decapoda	Pleocyemata (Caridea)	Palaemonidae	*Macrobrachium nipponense*	oriental river prawn	OB		(1) 16-cell stage	unnamed blastomere	LM, MM (*vasa in situ*, Vasa antibody), TEM	(Chen et al., 2021; Ma et al., 2019; Qiu et al., 2013)
Decapoda	Pleocyemata (Reptantia, Achelata)	Palinuridae	*Panulirus japonicus*	Japanese spiny lobster			(3) segmented embryo	ventral walls of mesodermal coelomic sacs in T1	LM	(Terao, 1929)
Anostraca	Artemiina	Artemiidae	*Artemia salina*				(3) nauplius hatchling	mesoderm of first trunk segment	LM	(Anderson, 1967)
Notostraca		Triopsidae	*Triops longicaudatus*				(3) fourth instar larva	on either side of the gut	LM	(Mitsumoto and Makioka, 2002)
Notostraca		Triopsidae	*Triops granarius*				(3) fourth instar larva	on either side of the gut	LM	(Mitsumoto and Makioka, 2003)
Spinicaudata		Limnadiidae	*Paralimnadia stanleyana*				(3) nauplius hatchling	mesoderm of first trunk segment	LM	(Anderson, 1967)
Ctenopoda		Holopediidae	*Holopedium gibberum*				(1) 16-cell stage	$D^{IV\,1}$ blastomere at the vegetal pole, which becomes the site of gastrulation	LM	(Baldass, 1937)
Ctenopoda		Sididae	*Penilia avirostris*				(3) late embryo	mesoderm on either side of intestine	LM	(Sudler, 1899)
Anomopoda		Macrothiricidae	*Lathonura rectirostris*				(2) gastrulation	four cells at blastopore lip	LM	(Grobben, 1879)
Anomopoda		Daphniidae	*Simocephalus vetulus*				(2) late blastoderm	posterior "ventral mass" on inside of blastoderm, later the site of gastrulation	LM	(Cannon, 1921)
Anomopoda		Daphniidae	*Daphnia magna*		OB		(1) 16-cell stage	unnamed blastomere	LM, MM (Vasa antibody)	(Sagawa et al., 2005)
Onychopoda		Cercopagididae	*Bythotrephes longimanus*	spiny water flea	OB		(1) 16-cell stage	one daughter cell of the dI micromere	LM	(Alwes and Scholtz, 2014)
Onychopoda		Polyphemidae	*Polyphemus pediculus*		OB		(1) 16-cell stage	one daughter cell of the dI micromere	LM, CL	(Kühn, 1913)
**HEXAPODA**										
Ametabola										
Poduromorpha		Neanuridae	*Anurida maritima*	seashore springtail			(3) segmented germ band	abdominal mesoderm	LM	(Claypole, 1898)
Poduromorpha		Onychiuridae	*Tetrodontophora bielanensis*	giant springtail	OB		(1) cleavage stage embryo	region of cortical cytoplasm in early-stage embryo	TEM	(Klag, 1982)
Symphypleona		Sminthuridae	*Allacma fusca*				(1) blastoderm-stage embryo	scattered in yolk	TEM	(Klag and Świątek, 1999)
Entomobryomorpha		Tomoceridae	*Tomocerus cuspidatus*				(1) cleavage-stage embryo	scattered in the yolk, inner mass	LM, TEM	(Tomizuka and Machida, 2015)
Entomobryomorpha		Tomoceridae	*Tomocerus ishibashii*				(1) blastoderm-stage embryo	center of yolk after blastoderm formation	LM	(Uemiya and Ando, 1991)
Archaeognatha		Machilidae	*Petrobius brevistylis*				(3) segmented germ band	dorsal coelomic sacs of T2–A1 mesoderm	LM	(Larink, 1969)
Zygentoma		Lepismatidae	*Lepisma saccharina*	silverfish			(2) early germ band	posterior end of germ band; author notes difficulty in following migration of putative germ cells	LM	(Heymons, 1897)
Zygentoma		Lepismatidae	*Thermobia domestica*	firebrat			(3) segmented germ band	dorsal coelomic sacs of abdominal mesoderm	LM	(Woodland, 1957)
Palaeoptera										
Odonata	Epiprocta	Epiophlebiidae	*Epiophlebia superstes*				(2) cellular blastoderm	posterior blastoderm; unclear whether these cells are the same cells that appear in the gonads later in embryogenesis	LM	(Ando, 1962)
Polyneoptera										
Dermaptera		Forficulidae	*Forficula auricularia*	common earwig			(2) cellular blastoderm	posterior blastoderm	LM	(Heymons, 1895)
Dermaptera		Labiduridae	*Labidura riparia*	shore earwig	OB		(1) syncytial blastoderm	posterior blastoderm	LM	(Singh, 1967)
Orthoptera	Ensifera	Gryllidae	*Oecanthus niveus*	narrow-winged tree			(3) late germ band	on either side of the dorsal vessel	LM	(Ayers, 1884)

Development • Supplementary information

				cricket						
Orthoptera	Ensifera	Gryllidae	*Acheta domesticus*	house cricket			(2) early germ band	posterior end of germ band	LM	(Heymons, 1895)
Orthoptera	Ensifera	Gryllidae	*Gryllus campestris*	European field cricket			(2) early germ band	posterior end of germ band	LM	(Heymons, 1895)
Orthoptera	Ensifera	Gryllidae	*Gryllus bimaculatus*	two-spotted field cricket		EX	(3) segmented germ band	abdominal mesoderm, before formation of coelomic sacs	LM, MM (Vasa and Piwi antibodies, many *in situ*), EX (genetic manipulation)	(Donoughe et al., 2014; Ewen-Campen et al., 2013a; Nakamura and Extavour, 2016)
Orthoptera	Ensifera	Tettigoniidae	*Conocephalus brevipennis*	short-winged meadow katydid			(3) late germ band	inner walls of coelomic sacs in A1–A6 mesoderm	LM	(Wheeler, 1893)
Orthoptera	Caelifera	Acrididae	*Melanoplus differentialis*	differential grasshopper			(2) early germ band	lateral margins of germ band, A1–A9	LM	(Nelsen, 1934)
Orthoptera	Caelifera	Acrididae	*Locusta migratoria*	migratory locust			(3) late germ band	dorsal coelomic sacs of A2–A5 mesoderm	LM	(Roonwal, 1937)
Orthoptera	Caelifera	Acrididae	*Schistocerca gregaria*	desert locust			(2) early germ band	lateral margins of abdominal germ band	LM, MM (Vasa antibody)	(Chang et al., 2002)
Blattodea	Solumblattodea	Blattidae	*Periplaneta orientalis*	oriental cockroach			(2) early germ band	posterior end of germ band	LM	(Heymons, 1895)
Blattodea	Blaberoidea	Ectobiidae	*Blattella germanica*	German cockroach			(2) early germ band	posterior end of germ band; author notes that putative germ cells are indistinguishable from mesodermal cells but identifies them as germ cells based on comparison with *Periplaneta orientalis*	LM	(Heymons, 1895)
Phasmatodea	Oriophasmata	Lonchodidae	*Carausius morosus*	common stick insect			(2) early germ band or (3) late germ band	(Hammerschmidt, 1910; Wiesmann, 1926) say posterior end of germ band; (Cavallin, 1970) says abdominal mesoderm	LM	(Cavallin, 1970; Hammerschmidt, 1910; Wiesmann, 1926)
Mantodea		Mantidae	*Rhombodera crassa*				(3) late germ band	coelomic sacs in A3–A5 mesoderm	LM	(Görg, 1959)
Condylognatha + Psocodea										
Thysanoptera	Tubulifera	Phlaeothripidae	*Haplothrips verbasci*		OB		(1) syncytial blastoderm	posterior blastoderm	LM	(Heming, 1979)
Thysanoptera	Tubulifera	Phlaeothripidae	*Bactrothrips brevitubus*		OB		(1) syncytial blastoderm	posterior blastoderm	LM	(Haga, 1985)
Hemiptera	Heteroptera (Pentatomomorpha)	Lygaeidae	*Oncopeltus fasciatus*	large milkweed bug			(2) cellular blastoderm	posterior blastoderm	LM, MM (Vasa antibody, many *in situ* hybridization)	(Butt, 1949; Ewen-Campen et al., 2013b; Kao et al., 2025)
Hemiptera	Heteroptera (Pentatomomorpha)	Blissidae	*Blissus leucopterus hirtus*	hairy chinch bug			(2) cellular blastoderm	posterior blastoderm	LM	(Choban and Gupta, 1972)
Hemiptera	Heteroptera (Pentatomomorpha)	Pyrrhocoridae	*Pyrrhochoris apterus*	European firebug			(3) segmented germ band	from the mesoderm in segments A1–A8	LM	(Seidel, 1924)
Hemiptera	Heteroptera (Cimicomorpha)	Reduviidae	*Rhodnius prolixus*	kissing bug			(2) cellular blastoderm	posterior blastoderm	LM, SEM	(Heming and Huebner, 1994; Kelly and Huebner, 1989; Mellanby, 1935)
Hemiptera	Sternorrhynca	Monophlebidae	*Icerya purchasi*	cottony cushion scale			(2) cellular blastoderm	posterior blastoderm	LM	(Shinji, 1919)
Hemiptera	Sternorrhynca	Pseudococcidae	*Pseudococcus citri*	citrus mealybug			(2) cellular blastoderm	posterior blastoderm	LM	(Schrader, 1922)
Hemiptera	Sternorrhynca	Aphididae	*Acyrthosiphon pisum*	pea aphid	OB		(2) cellular blastoderm	posterior blastoderm	LM, MM (Vasa, Nanos antibody, *vasa in situ*)	(Chang et al., 2006; Chang et al., 2007; Lin et al., 2014; Miura et al., 2003)
Psocodea	Psocomorpha	Archipsocidae	*Archipsocus fernandi*				(2) cellular blastoderm	posterior blastoderm	LM	(Fernando, 1934)
Psocodea	Troctomorpha	Liposcelididae	*Liposcelis divergens*	book louse			(2) cellular blastoderm	posterior blastoderm	LM	(Goss, 1952; Goss, 1953)
Holometabola										
Hymenoptera	Apocrita (Aculeata)	Formicidae	*Lasius niger*	black garden ant	OB		(1) cellular blastoderm	posterior pole of blastoderm	LM, MM (Vasa antibody, *nanos, oskar in situ*)	(Rafiqi et al., 2020)
Hymenoptera	Apocrita (Aculeata)	Formicidae	*Camponotus floridanus*	Florida carpenter ant	OB		(1) cellular blastoderm	posterior pole of blastoderm	LM, MM (Vasa antibody, *nanos, oskar in situ*)	(Rafiqi et al., 2020)
Hymenoptera	Apocrita (Aculeata)	Formicidae	*Messor pergandei*	black harvester ant	OB		(1) cellular blastoderm	posterior pole of blastoderm	LM, MM (*oskar, vasa, nanos in situ*)	(Khila and Abouheif, 2010; Lynch et al., 2011)
Hymenoptera	Apocrita (Aculeata)	Apidae	*Apis mellifera*	western honeybee			(3) segmented germ band	genital ridges in A3–A6 mesoderm	LM, SEM, MM (*vasa, nanos in situ*)	(Dearden, 2006; Fleig and Sander, 1986; Nelson, 1915)
Hymenoptera	Apocrita (Parasitoida)	Pteromalidae	*Nasonia vitripennis*	jewel wasp	EX		(1) syncytial blastoderm (nuclear cycle 8)	pole cells at posterior pole of blastoderm	LM, MM (*nanos, oskar in situ*), EX (genetic manipulation), TS	(Bull, 1982; Lynch and Desplan, 2010; Lynch et al., 2011; Quan et al., 2019)
Hymenoptera	Apocrita (Parasitoida)	Trichogrammatidae	*Trichogramma evanescens*		OB		(1) syncytial blastoderm	pole cells at posterior pole of blastoderm	LM	(Gatenby, 1917; Gatenby, 1918)
Hymenoptera	Apocrita (Parasitoida)	Encyrtidae	*Copidosoma floridanum*		EX		(1) 4-cell stage	B4 blastomere	LM, MM (Vasa *in situ* and antibody), EX (cell ablation)	(Donnell et al., 2004; Grbic', 2003; Zhurov et al., 2004)

Hymenoptera	Apocrita (Parasitoida)	Braconidae	*Macrocentrus cingulum*		OB		(2) cellular blastoderm	inside the posterior blastocoel	LM, MM (Vasa antibody)	(Grbic', 2003; Sucena et al., 2014)
Hymenoptera	Apocrita (Parasitoida)	Braconidae	*Coeloides vancouverensis*		OB		(1) syncytial blastoderm	pole cells at posterior pole of blastoderm	LM	(Ryan, 1963)
Hymenoptera	Apocrita (Parasitoida)	Braconidae	*Habrobracon juglandis*		OB		(1) syncytial blastoderm	pole cells at posterior pole of blastoderm	LM	(Amy, 1961)
Hymenoptera	Apocrita (Parasitoida)	Ichneumonidae	*Cosmoconus meridionator*		OB		(1) syncytial blastoderm	pole cells at posterior pole of blastoderm	TEM	(Klag and Bilinski, 1993)
Hymenoptera	Apocrita (Parasitoida)	Ichneumonidae	*Lissonota catenator*		OB		(1) syncytial blastoderm	pole cells at posterior pole of blastoderm	TEM	(Klag and Bilinski, 1993)
Hymenoptera	Apocrita (Parasitoida)	Ichneumonidae	*Pimpla turionellae*		EX	EX	(1) syncytial blastoderm	pole cells at posterior pole of blastoderm (note: evidence that germ line can also regenerate)	LM, EX (damage cytoplasm)	(Achtelig and Krause, 1971; Bronskill, 1959; Meng, 1968)
Hymenoptera	Eusymphyta	Tenthredinidae	*Nematus ribesii*	gooseberry sawfly			(3) segmented germ band	posterior midgut rudiment	LM	(Shafiq, 1954)
Hymenoptera	Eusymphyta	Tenthredinidae	*Athalia rosae*	turnip sawfly			(1) syncytial blastoderm	pole cells at posterior pole of blastoderm	LM, MM (Vasa antibody)	(Nakao et al., 2006)
Raphidioptera		Inocelliidae	*Inocellia japonica*				(1) syncytial blastoderm	pole cells at posterior pole of blastoderm	LM, SEM	(Tsutsumi and Machida, 2006)
Megaloptera		Sialidae	*Sialis mitsuhashii okamoto*	alderfly			(2) late blastoderm	posterior end of ventral plate	TEM	(Suzuki et al., 1981)
Strepsiptera		Stylopidae	*Stylops spp.*				(3) extended germ band	posterior end of the germ band	LM	(Noskiewicz and Poluszyński, 1927)
Coleoptera	Adephaga	Carabidae	*Carabus insulicola*				??	no pole cells observed	LM, SEM	(Kobayashi et al., 2013)
Coleoptera	Adephaga	Gyrinidae	*Dineutus mellyi*	whirligig beetle			??	no pole cells observed	LM, SEM	(Komatsu and Kobayashi, 2012)
Coleoptera	Adephaga	Dytiscidae	*Hydaticus pacificus*	diving beetle			??	no pole cells observed	LM, SEM	(Niikura et al., 2017)
Coleoptera	Polyphaga (Elateroidea)	Lampyridae	*Nipponoluciola cruciata*				??	no pole cells observed	LM	(Kobayashi and Ando, 1985)
Coleoptera	Polyphaga (Elateroidea)	Lampyridae	*Pyrocoelia rufa*				??	no pole cells observed	LM	(Kobayashi et al., 2006)
Coleoptera	Polyphaga (Hydrophiloidea)	Hydrophilidae	*Hydrophilus piceus*	great silver water beetle			(3) segmented germ band	from the walls of the coelomic sacs of abdominal mesoderm	LM	(Heider, 1889)
Coleoptera	Polyphaga (Scarabaeoidea)	Passalidae	*Odontotaenius disjunctus*	patent-leather beetle, horned passalus			(3) late germ band	dorsal side of abdominal germ band, near A6–A7	LM	(Krause, 1947)
Coleoptera	Polyphaga (Scarabaeoidea)	Scarabaeidae	*Phyllophaga fervida*				(2) cellular blastoderm	posterior blastoderm	LM	(Luginbill, 1953)
Coleoptera	Polyphaga (Bostrichoidea)	Dermestidae	*Dermestes frischii*		OB		(2) cellular blastoderm	posterior blastoderm	LM	(Küthe, 1966)
Coleoptera	Polyphaga (Tenebrionoidea)	Meloidae	*Lytta viridana*	blister beetle			(2) cellular blastoderm	posterior blastoderm	LM	(Church and Rempel, 1971; Rempel and Church, 1969)
Coleoptera	Polyphaga (Tenebrionoidea)	Tenebrionidae	*Tenebrio molitor*	yellow mealworm beetle			(2) cellular blastoderm	posterior blastoderm	LM	(Ullmann, 1964)
Coleoptera	Polyphaga (Tenebrionoidea)	Tenebrionidae	*Tribolium confusum*	confused flour beetle			(2) cellular blastoderm	posterior blastoderm	LM	(Hodson, 1934; Stanley and Grundmann, 1970)
Coleoptera	Polyphaga (Tenebrionoidea)	Tenebrionidae	*Tribolium castaneum*	red flour beetle		EX	(2) cellular blastoderm	posterior blastoderm	LM, MM (*vasa in situ*), EX (genetic manipulation)	(Ansari et al., 2018; Schröder, 2006)
Coleoptera	Polyphaga (Curculionidea)	Curculionidae	*Sphenophorus callosus*	southern corn billbug	EX		(1) syncytial blastoderm	pole cells at posterior pole of blastoderm	LM, EX (cytoplasmic cauterization)	(Hegner, 1911; Hegner, 1914; Wray, 1937)
Coleoptera	Polyphaga (Curculionidea)	Curculionidae	*Otiorhynchus ligustici*	alfalfa snout beetle	OB		(1) syncytial blastoderm	pole cells at posterior pole of blastoderm	LM	(Butt, 1936)
Coleoptera	Polyphaga (Curculionidea)	Curculionidae	*Sitophilus oryzae*	rice weevil			(1) syncytial blastoderm	pole cells at posterior pole of blastoderm	LM	(Tiegs and Murray, 1938)
Coleoptera	Polyphaga (Chrysomeloidea)	Chrysomelidae	*Calligrapha multipunctata*	common willow calligrapha	EX		(1) syncytial blastoderm	pole cells at posterior pole of blastoderm	LM, EX (cytoplasmic removal)	(Hegner, 1908; Hegner, 1909a; Hegner, 1909b)
Coleoptera	Polyphaga (Chrysomeloidea)	Chrysomelidae	*Leptinotarsa decemlineata*	Colorado potato beetle	EX		(1) syncytial blastoderm	pole cells at posterior pole of blastoderm	LM, EX (cytoplasmic cauterization/removal)	(Hegner, 1908; Hegner, 1911; Hegner, 1914)
Coleoptera	Polyphaga (Chrysomeloidea)	Chrysomelidae	*Callosobruchus maculatus*	cowpea weevil	EX		(1) syncytial blastoderm	pole cells at posterior pole of blastoderm	LM, MM (*oskar, vasa, tudor in situ*), EX (UV irradiation)	(Brauer, 1925; Brauer, 1949; Quan, 2018)
Coleoptera	Polyphaga (Chrysomeloidea)	Chrysomelidae	*Acanthoscelides obtectus*	bean weevil	OB		(1) syncytial blastoderm (nuclear cycle 8)	pole cells at posterior pole of blastoderm	LM, MM (*vasa in situ*), TEM	(Jung, 1966; Lynch et al., 2011)

Coleoptera	Polyphaga (Chrysomeloidea)	Chrysomelidae	*Euryope terminalis*	milkweed leaf beetle	OB		(1) syncytial blastoderm	pole cells at posterior pole of blastoderm	LM	(Paterson, 1931)
Coleoptera	Polyphaga (Chrysomeloidea)	Chrysomelidae	*Platycorynus compressicornis*		OB		(1) syncytial blastoderm	pole cells at posterior pole of blastoderm	LM	(Paterson, 1935)
Trichoptera	Annulipalpia	Stenopsychidae	*Stenopsyche griseipennis*				(3) segmented germ band	genital ridges in A2–A7 mesoderm	LM	(Miyakawa, 1974)
Lepidoptera	Micropterigoidea	Micropterigidae	*Neomicropteryx nipponensis*				(3) segmented germ band	mediodorsal coelomic sacs in A5 mesoderm	LM	(Kobayashi and Ando, 1984)
Lepidoptera	Glossata (Hepialoidea)	Hepialidae	*Endoclita signifer*				(2) early germ band	posterior germ band	LM	(Ando and Tanaka, 1980)
Lepidoptera	Apoditrysia (Tortrichoidea)	Tortricidae	*Epiphyas postvittana*	light brown apple moth			(2) early germ band	between germ band and yolk	LM	(Anderson and Wood, 1968)
Lepidoptera	Obtectomera (Papilionoidea)	Nymphalidae	*Euvanessa (Nymphalis) antiopa*	mourning cloak			(2) early germ band	midline of ventral plate, about one-third length from the posterior	LM	(Woodworth, 1889)
Lepidoptera	Obtectomera (Papilionoidea)	Nymphalidae	*Pararge aegeria*	speckled wood butterfly	OB		(2) cellular blastoderm	ventral blastoderm	LM, MM (*nanos in situ*), TS	(Carter et al., 2013; Carter et al., 2015)
Lepidoptera	Obtectomera (Papilionoidea)	Pieridae	*Pieris rapae*	cabbage white, cabbage butterfly			(3) segmented germ band	coelomic sacs of A4–A5 mesoderm	LM	(Eastham, 1931)
Lepidoptera	Obtectomera (Papilionoidea)	Papilionidae	*Parnassius glacialis*	glacial Apollo, Japanese clouded Apollo			(2) late cellular blastoderm/early germ band	midline of ventral plate, about one-third length from the posterior	LM	(Tanaka, 1987)
Lepidoptera	Obtectomera (Papilionoidea)	Papilionidae	*Luehdorfia japonica*	Gifu butterfly			(2) early germ band	midline of germ band, about one-third length from the posterior	LM	(Tanaka, 1987)
Lepidoptera	Obtectomera (Papilionoidea)	Papilionidae	*Byasa alcinous alcinous*	Chinese windmill			(2) early germ band	midline of germ band, about one-third length from the posterior	LM	(Tanaka, 1987)
Lepidoptera	Obtectomera (Gelechioidea)	Gelechiidae	*Pectinophora gossypiella*	pink bollworm	OB		(2) cellular blastoderm	posterior, ventral blastoderm	TEM	(Berg and Gassner, 1978)
Lepidoptera	Macroheterocera (Noctuoidea)	Amatidae	*Amata fortunei*	white-spotted moth			(2) early germ band	midline of germ band, near the center	LM	(Tanaka, 1985)
Lepidoptera	Macroheterocera (Noctuoidea)	Noctuidae	*Helicoverpa zea*	corn earworm			(2) early germ band	midline of germ band, near the center	LM	(Presser and Rutschky, 1957)
Lepidoptera	Macroheterocera (Noctuoidea)	Arctiinae	*Spilosoma virginica*	Virginian tiger moth			(3) late germ band	coelomic sacs in abdominal mesoderm	LM	(Johannsen, 1929)
Lepidoptera	Macroheterocera (Bombycoidea)	Bombycidae	*Bombyx mori*	domestic silk moth	EX	EX	(2) cellular blastoderm	midline of ventral plate, about one-third length from the posterior		(Miya, 1958; Nakao, 1999; Nakao and Takasu, 2019)
Lepidoptera	Macroheterocera (Bombycoidea)	Saturniidae	*Antheraea pernyi*	Chinese (oak) tussar moth			(3) segmented germ band	mesodermal coelomic sacs of all abdominal segments	LM	(Saito, 1937)
Mecoptera		Panorpidae	*Panorpa pryeri*		OB		(2) early germ band	posterior end of germ band	LM	(Suzuki, 1990)
Mecoptera		Panorpodidae	*Panorpodes paradoxa*				(2) early germ band	posterior end of germ band	LM	(Suzuki, 1990)
Mecoptera		Bittacidae	*Bittacus laevipes*				(2) early germ band	posterior end of germ band	LM	(Suzuki, 1990)
Mecoptera		Boreidae	*Boreus westwoodi*				(2) early germ band	posterior end of germ band	LM	(Suzuki, 1990)
Siphonaptera		Pulicidae	*Ctenocephalides felis*	cat flea			(1) syncytial blastoderm (nuclear cycle 6)	pole cells at posterior pole of blastoderm	LM	(Kessel, 1939)
Diptera	Psychodomorpha	Psychodidae	*Phlebotomus papatasi*	sand fly	OB		(1) syncytial blastoderm	pole cells at posterior pole of blastoderm	LM	(Abbassy et al., 1995a; Abbassy et al., 1995b; Abbassy et al., 1995c)
Diptera	Psychodomorpha	Psychodidae	*Clogmia albipunctata*	moth fly		EX	(2) cellular blastoderm	posterior blastoderm; no morphologically distinct pole cells	SEM, LM, MM (Vasa antibody, *nanos in situ*), EX (genetic manipulation)	(Jiménez-Guri et al., 2014; Yoon et al., 2019)
Diptera	Culicomorpha (Chironomoidea)	Simuliidae	*Simulium pictipes*	black fly	OB		(1) syncytial blastoderm	pole cells at posterior pole of blastoderm	LM	(Gambrell, 1933)
Diptera	Culicomorpha (Chironomoidea)	Chironomidae	*Chironomus sp.*				(1) syncytial blastoderm	pole cells at posterior pole of blastoderm	LM	(Klomp et al., 2015; Weissmann, 1863)
Diptera	Culicomorpha (Chironomoidea)	Chironomidae	*Chironomus confinis*	nonbiting midge	OB		(1) syncytial blastoderm	pole cells at posterior pole of blastoderm	LM	(Hegner, 1914)
Diptera	Culicomorpha (Chironomoidea)	Chironomidae	*Smittia sp.*		OB		(1) syncytial blastoderm	pole cells at posterior pole of blastoderm	LM, TEM	(Zissler and Sander, 1973)
Diptera	Culicomorpha (Culicoidea)	Culicidae	*Aedes aegypti*	yellow fever mosquito	OB		(1) syncytial blastoderm	pole cells at posterior pole of blastoderm	LM, MM (*oskar in situ*)	(Juhn and James, 2006; Raminani and Cupp, 1975)
Diptera	Culicomorpha (Culicoidea)	Culicidae	*Culex quinquefasciatus*	southern house mosquito	OB		(1) syncytial blastoderm	pole cells at posterior pole of blastoderm	LM, MM (*nanos*, *oskar in situ*)	(Davis, 1967; Juhn et al., 2008)

Diptera	Culicomorpha (Culicoidea)	Culicidae	*Culiseta inornata*	winter marsh mosquito	OB		(1) syncytial blastoderm	pole cells at posterior pole of blastoderm	LM	(Harber and Mutchmor, 1970)
Diptera	Culicomorpha (Culicoidea)	Culicidae	*Anopheles gambiae*	African malaria mosquito	OB		(1) syncytial blastoderm (nuclear cycle 9)	pole cells at posterior pole of blastoderm	LM, MM (*oskar in situ*)	(Juhn and James, 2006)
Diptera	Bibionomorpha	Cecidomyiidae	*Miastor metraloas*	gall midge	OB		(1) syncytial blastoderm (nuclear cycle 3)	pole cells at posterior pole of blastoderm	LM, TEM	(Hegner, 1914; Mahowald, 1975)
Diptera	Bibionomorpha	Cecidomyiidae	*Mayetiola destructor*	Hessian fly			(1) syncytial blastoderm (nuclear cycle 5)	pole cells at posterior pole of blastoderm	LM	(Metcalfe, 1935)
Diptera	Bibionomorpha	Sciaridae	*Bradysia coprophila*	black fungus gnat	OB		(1) syncytial blastoderm (nuclear cycle 5)	pole cells at posterior pole of blastoderm	LM	(DuBois, 1932; de Saint Phalle and Sullivan, 1996)
Diptera	Bibionomorpha	Sciaridae	*Sciara sp.*		OB		(1) syncytial blastoderm (nuclear cycle 5)	pole cells at posterior pole of blastoderm	LM	(Butt, 1934)
Diptera	Cyclorrapha (Platypezoidea)	Phoridae	*Megaselia abdita*	scuttle fly	OB		(1) syncytial blastoderm (nuclear cycle 9)	pole cells at posterior pole of blastoderm	SEM, LM, MM (Vasa antibody)	(Wotton et al., 2014)
Diptera	Schizophora (Tephritoidea)	Tephritidae	*Bactrocera tryoni*	Queensland fruit fly	OB		(1) syncytial blastoderm (nuclear cycle 10)	pole cells at posterior pole of blastoderm	LM	(Anderson, 1962)
Diptera	Schizophora (Ephydroidea)	Drosophilidae	*Drosophila melanogaster*	common fruit fly, vinegar fly	EX		(1) syncytial blastoderm (nuclear cycle 9)	pole cells at posterior pole of blastoderm	LM, EM, MM (many), EX (genetic manipulation, cytoplasmic transplant)	(Counce, 1963; Ephrussi and Lehmann, 1992; Huettner, 1923; Illmensee and Mahowald, 1974; Mahowald, 1968)
Diptera	Schizophora (Ephydroidea)	Drosophilidae	*Drosophila hydei*		OB		(1) syncytial blastoderm	pole cells at posterior pole of blastoderm	LM, TEM	(Counce, 1963; Mahowald, 1968)
Diptera	Schizophora (Ephydroidea)	Drosophilidae	*Drosophila willistoni*		OB		(1) syncytial blastoderm	pole cells at posterior pole of blastoderm	LM, TEM	(Counce, 1963; Mahowald, 1968)
Diptera	Schizophora (Ephydroidea)	Drosophilidae	*Drosophila immigrans*		EX		(1) syncytial blastoderm	pole cells at posterior pole of blastoderm	TEM, EX (cytoplasmic transplant)	(Mahowald, 1968; Mahowald et al., 1976)
Diptera	Schizophora (Ephydroidea)	Drosophilidae	*Drosophila busckii*		OB		(1) syncytial blastoderm	pole cells at posterior pole of blastoderm	LM	(Counce, 1963)
Diptera	Schizophora (Ephydroidea)	Drosophilidae	*Drosophila virilis*		OB		(1) syncytial blastoderm	pole cells at posterior pole of blastoderm	LM, MM (Vasa, Oskar antibody, many *in situ*)	(Counce, 1963; Rivard et al., 2025)
Diptera	Schizophora (Ephydroidea)	Drosophilidae	*Drosophila americana*		OB		(1) syncytial blastoderm	pole cells at posterior pole of blastoderm	LM	(Counce, 1963)
Diptera	Schizophora (Ephydroidea)	Drosophilidae	*Drosophila repleta*		OB		(1) syncytial blastoderm	pole cells at posterior pole of blastoderm	LM	(Counce, 1963)
Diptera	Schizophora (Ephydroidea)	Drosophilidae	*Drosophila gibberosa*		OB		(1) syncytial blastoderm	pole cells at posterior pole of blastoderm	LM	(Counce, 1963)
Diptera	Schizophora (Ephydroidea)	Drosophilidae	*Drosophila funebris*		OB		(1) syncytial blastoderm	pole cells at posterior pole of blastoderm	LM	(Counce, 1963)
Diptera	Schizophora	Hippoboscidae	*Melophagus ovinus*	sheep ked, sheep tick	OB		(1) syncytial blastoderm	pole cells at posterior pole of blastoderm	LM	(Lassmann, 1936)
Diptera	Schizophora	Muscidae	*Stomoxys calcitrans*	stable fly			(1) syncytial blastoderm	pole cells at posterior pole of blastoderm	LM, TEM, SEM	(Ajidagba et al., 1983)
Diptera	Schizophora	Muscidae	*Musca domestica*	house fly	OB		(1) syncytial blastoderm (nuclear cycle 6)	pole cells at posterior pole of blastoderm	LM	(West et al., 1968)
Diptera	Schizophora (Oestroidea)	Calliphoridae	*Calliphora vicina*	blowfly	OB		(1) syncytial blastoderm	pole cells at posterior pole of blastoderm	LM	(Hegner, 1914)
Diptera	Schizophora (Oestroidea)	Calliphoridae	*Phormia regina*	black blowfly	OB		(1) syncytial blastoderm	pole cells at posterior pole of blastoderm	LM	(Auten, 1934)
Diptera	Schizophora (Oestroidea)	Calliphoridae	*Lucilia sericata*	sheep blowfly	OB		(1) syncytial blastoderm (nuclear cycle 10)	pole cells at posterior pole of blastoderm	LM	(Davis, 1967; Fish, 1947a; Fish, 1947b; Fish, 1949; Fish, 1952)

Table S2. Studies used for phylogram and constraint tree construction. Clade: clade extracted from the cited study; Resolved rank: lowest taxonomic rank resolved in our phylogram from the study.

Reference	Clade	Resolved rank	Note
(Howard et al., 2022)	Panarthropoda	Phylum	
(Baker et al., 2021)	Onychophora	Family	
(Giribet, 2018)	Chelicerata	Order	Euchelicerate relationships outside of Arachnopulmonata were left as a polytomy to reflect the continued uncertainty in chelicerate phylogeny (Giribet, 2018; Sharma and Gavish-Regev, 2025).
(Sabroux et al., 2023)	Pantopoda	Family	
(Fernández et al., 2018)	Myriapoda	Class	
(Bernot et al., 2023)	Pancrustacea	Family, Order, Subphylum	Lower-rank constraints for Hexapoda, Decapoda, Isopoda, Amphipoda, Diplostraca, and Copepoda were substituted from other studies.
(Wolfe et al., 2019)	Decapoda	Family	
(Thorpe, 2024)	Isopoda	Family	
(Copilaş-Ciocianu et al., 2020)	Amphipoda	Family	
(Sun and Cheng, 2023)	Diplostraca	Family	
(Eyun, 2017)	Copepoda	Family	
(Yu et al., 2024)	Collembola	Order	
(Misof et al., 2014)	Insecta	Order	All order-level constraints were followed except for Mecoptera, which was constrained as a polytomy with Siphonaptera to reflect recent evidence supporting the paraphyly of Mecoptera (Meusemann et al., 2020; Tihelka et al., 2020).
(Chang et al., 2020)	Orthoptera	Family	
(Song et al., 2024)	Hemiptera	Family	
(Blaimer et al., 2023)	Hymenoptera	Family	(Pteromalidae + Encytridae + Trichogrammatidae) and (Tenthredinidae + Athaliidae) were left as polytomies in the constraint tree due to non-monophyly.
(Zhang et al., 2018)	Coleoptera	Family	

Development • Supplementary information

(Nie et al., 2020)	Chrysomelidae	Subfamily	
(Kawahara et al., 2019)	Lepidoptera	Family	
(Tihelka et al., 2020)	Siphonaptera + Mecoptera	Family	
(Wiegmann et al., 2011)	Diptera	Family	
(Suvorov et al., 2022)	Drosophilidae	Species	
(daSilva et al., 2020)	Culicidae	Genus	

Table S3. Hypothesized transitions in germ cell specification in Panarthropoda. Clade name = monophyletic group in whose ancestral lineage the transition is specified to have occurred; Node_number = node labels in Figures S1 and S2; Transition = gain of germ plasm (GP) or reversion to induction (IND); Parsimony = support from maximum parsimony (Figure S1); Likelihood = support from maximum likelihood (Figure S2).

See Table S3 at https://github.com/rishabhrajkapoor/panarthropoda_gc_specification_evolution, under supplementary_tables, commit ID ff9945c.

Table S4. Genomic and transcriptomic datasets used for phylogenetic inference.

We were unable to find sequence data for species in the “sub_species” columns, and instead substituted the genomes or transcriptomes for species in the same taxonomic family. Species in the “sub_species” columns are replaced by “iq_tree_label” in trees.

See Table S4 at https://github.com/rishabhrajkapoor/panarthropoda_gc_specification_evolution, under supplementary_tables, commit ID ff9945c.

Table S5. Literature-derived fossil calibrations. Node = clade name calibrated by fossil bounds; Spanning set = list of two taxa spanning the full clade in our tree; Min = minimum node age (millions of years) used for soft fossil constraints in MCMCTree; Max = maximum node age used for soft fossil constraints in MCMCTree. In the Node column, "+" indicates a node representing the common ancestor of two monophyletic lineages. Note that Mecoptera was treated as paraphyletic in this study (see Table S2), such that the "Siphonaptera and Mecoptera" node represents the common ancestor of all Mecoptera and Siphonaptera.

Node	Spanning set	Min	Max	Reference
Panarthropoda	Parachela, *Drosophila_virilis*	528.8	636.1	(Howard et al., 2022)
Chelicerata	Nymphonidae, *Parasteatoda_tepidariorum*	509.0	636.1	(Howard et al., 2022)
Euchelicerata	Pseudoscorpiones, *Parasteatoda_tepidariorum*	500.5	636.1	(Howard et al., 2022)
Arachnopulmonata	Euscorpiidae, *Parasteatoda_tepidariorum*	435.2	514.0	(Howard et al., 2022)
Holometabola	Liposcelididae, *Camponotus_floridanus*	306.9	450.0	(Misof et al., 2014)
Pterygota	*Epiophlebia_superstes, Drosophila_virilis*	324.0	450.0	(Misof et al., 2014)
Dicondylia	*Thermobia_domestica, Drosophila_virilis*	411.5	450.0	(Misof et al., 2014)
Thysanoptera + Hemiptera	Phlaeothripidae, *Rhodnius_prolixus*	207.0	450.0	(Misof et al., 2014)
Hymenoptera	Tenthredinidae, *Camponotus_floridanus*	221	450.0	(Misof et al., 2014)
Formicidae + Apidae	*Camponotus_floridanus, Apis_mellifera*	89.8	221.0	(Misof et al., 2014)
Gyrinidae + Carabidae	*Gyrinus_marinus, Elaphrus_aureus*	205.6	306.0	(Misof et al., 2014)
Tenebrionidae + Curculionidae	*Sitophilus_oryzae, Tribolium_castaneum*	207.0	306.0	(Misof et al., 2014)
Lepidoptera	Hepialidae, *Bombyx_mori*	112.0	306.9	(Misof et al., 2014)
Papilionoidea	Papilionidae, *Pararge_aegeria*	34.1	112.0	(Misof et al., 2014)
Siphonaptera and Mecoptera	*Ctenocephalides_felis, Boreidae*	160.5	306.9	(Misof et al., 2014)
Diptera	*Phlebotomus_papatasi, Drosophila_virilis*	230.0	306.9	(Wiegmann et al., 2011)
Schizophora	*Bactrocera_tryoni, Drosophila_virilis*	64.0	230.0	(Wiegmann et al., 2011)
Sciaroidea	*Bradysia_coprophila, Mayetiola_destructor*	180.0	230.0	(Wiegmann et al., 2011)
Arthropoda	*Limulus_polyphemus, Drosophila_virilis*	514.0	636.1	(Bernot et al., 2023)
Branchiopoda	Triopsidae, *Daphnia_magna*	405.0	521.0	(Bernot et al., 2023)
Collembola	Tomoceridae, *Tetrodontophora_bielanensis*	405.0	521.0	(Bernot et al., 2023)
Peracarida	Gammaridae, *Armadillidium_nasatum*	358.2	521.0	(Bernot et al., 2023)

Development • Supplementary information

Table S6. phytools model fits for maximum likelihood ancestral state reconstruction. SYM = symmetric substitution rates; ARD = asymmetric substitution rates; SYM.G = symmetric substitution rates with gamma heterogeneity; ARD.G = asymmetric substitution rates with gamma heterogeneity; HRM2 = hidden rates model with symmetric substitution rates and two hidden rate categories. log(L) = log likelihood score; d.f. = degrees of freedom (number of model parameters); AIC = Akaike information criterion; Weight = model importance via ANOVA.

Model	log(L)	d.f.	AIC	Weight
SYM	-68.10199	1	138.204	0.0065576866
ARD	-67.93943	2	139.879	0.0028382863
SYM.G	-62.41206	2	128.824	0.7137752031
ARD.G	-62.36246	3	130.725	0.2759361780
HRM2	-67.09620	4	142.192	0.0008926461

Table S7. Marginal probabilities at select nodes. Probabilities of germ plasm nodes at select ancestors derived via maximum likelihood. Node_number = labels at internal nodes of Figures S1 and S2; IND = induction; GP = germ plasm.

Clade	Node_number	P_IND	P_GP
Panarthropoda	1	0.994	0.006
Arthropoda	2	1.000	0.000
Chelicerata	3	1.000	0.000
Myriapoda	4	0.994	0.006
Pancrustacea	5	1.000	0.000
Decapoda	6	0.119	0.881
Hyalidae + Talitridae	7	0.084	0.916
Poduromorpha + Symphypleona	8	0.668	0.332
Collembola	9	0.987	0.013
Insecta	10	0.999	0.001
Holometabola	11	0.806	0.194
Hymenoptera	12	0.283	0.717
Coleoptera	13	0.986	0.014
Chrysomelidae + Curculionidae	14	0.120	0.880
Lepidoptera	15	0.996	0.004
Mecoptera + Siphonaptera	16	0.518	0.482
Antliophora (Diptera + Mecoptera + Siphonaptera)	17	0.532	0.468
Diptera	18	0.385	0.615

Table S8. Results of Pagel's test of correlated rates in germ band type and germ cell specification mode.

Model	log(L)	AIC	p-value
Independent	-79.52302	167.0460	n/a
Bidirectional	-65.03560	146.0712	7.90998e-06
GC dependent	-69.61696	151.2339	4.98718e-05
GB dependent	-68.75534	149.5107	2.10696e-05

Table S9. Results of search for *oskar* orthologs. BUSCO scores may differ from Table S4 for the seven unannotated genomes used in our analysis (*Clogmia albipunctata, Mayetiola destructor, Macrocentus cingulum, Epiphyas postvittana, Nymphalis antiopa, Pyrrhocoris apterus, Tribolium confusum*), as BUSCO scores and *oskar* presence are determined from AUGUSTUS genome annotations. "Blondel_oskar_count" column indicates the total number of *oskar* orthologs previously identified across all datasets from this species (Blondel et al., 2021). Empty cells indicate species that were not analyzed in our previous study on *oskar* ortholog evolution (Blondel et al., 2021). "oskar_present" column indicates that *oskar* was detected in the current analysis and/or in our previous work (Blondel et al., 2021). "data_type" indicates whether the protein predictions were obtained directly from protein fasta files on NCBI ("NCBI-annotated genome"), from TransDecoder run on transcriptome shotgun assembly nucleotide sequences ("TSA"), or from *ab initio* annotations generated with AUGUSTUS ("AUGUSTUS-annotated genome"). We were unable to find sequence data for species in the "sub_species" column, and instead substituted the genomes/transcriptomes for species in the same taxonomic family. The "oskar_accession" columns provide either NCBI protein accessions for NCBI-annotated genomes ("https://www.ncbi.nlm.nih.gov/protein/"), TSA nucleotide accessions for TSAs ("https://www.ncbi.nlm.nih.gov/genbank/tsa/"), or AUGUSTUS-generated accessions for AUGUSTUS-annotated genomes. All Oskar protein sequences are available in oskar_proteins.fa.

See Table S9 at https://github.com/rishabhrajkapoor/panarthropoda_gc_specification_evolution, under supplementary tables, commit ID ff9945c.

Development • Supplementary information
